# Evaluation of appropriate transmission-based precautions for non-SARS-CoV-2 respiratory viruses

**DOI:** 10.1017/ash.2023.472

**Published:** 2023-10-31

**Authors:** Cassandra L. Falk, Lisa E. Dumkow, Abigail C. Geyer, Kyle J. Schmidt, Nnaemeka E. Egwuatu, Jennifer M. Langholz, Andrew P. Jameson

**Affiliations:** 1 Department of Pharmacy, Trinity Health Grand Rapids, Grand Rapids, MI, USA; 2 Ferris State University, College of Pharmacy, Grand Rapids, MI, USA; 3 Division of Infection Control, Trinity Health Grand Rapids, Grand Rapids, MI, USA; 4 Division of Infectious Disease, Trinity Health Grand Rapids, Grand Rapids, MI, USA; 5 Department of Medicine, Michigan State College of Human Medicine, Grand Rapids, MI, USA

## Abstract

Appropriateness of transmission-based precautions after positive result for a non-SARS-CoV-2 virus was evaluated. Most patients (77.2%) lacked appropriate precautions within 3 hours of virus detection; 36.9% remained without appropriate precautions during their stay. With recent cessation of universal masking, adherence to infection control best practices is needed to optimize safety.

## Introduction

The utilization of rapid multiplex PCR (mPCR) testing to detect infectious respiratory pathogens peaked during the emergence of the Severe Acute Respiratory Syndrome Coronavirus 2 (SARS-CoV-2) pandemic. Before the pandemic, several studies evaluated viral upper respiratory mPCR testing’s impact on antimicrobial prescribing, hospital length of stay, and mortality showing mixed results.^1–4^ Few authors have evaluated the test’s impact on infection control (IC) practices including appropriateness and timing of precautions to protect healthcare team members and patients within the emergency department (ED) and inpatient settings.^5–9^ The purpose of this study was to evaluate the appropriateness of transmission-based precautions (TBPs) following a positive rapid upper respiratory mPCR result for a non-SARS-CoV-2 virus.

## Materials and methods

This Institutional Review Board-approved, retrospective cohort study was conducted at a 350-bed community teaching hospital in West Michigan. Adult patients with an upper respiratory mPCR sample obtained in the ED and positive for a viral pathogen between November 1, 2021, and October 31, 2022, were screened for inclusion. Patients with SARS-CoV-2 virus, mPCR testing performed after ED arrival, or without documented respiratory symptoms were excluded.

### Institution infection control procedures

Our institution utilized the BioFire Respiratory 2.1 mPCR (BioFire Diagnostics, Salt Lake City, UT) during the study period. This rapid mPCR tests for 22 targets, including 17 non-SARS-CoV-2 viral pathogens. Rapid upper respiratory mPCR testing requires a provider order and results are available in under 1 hour. When a viral pathogen is identified, a passive best practice alert (BPA) fires within the electronic health record (EHR) to notify the treatment team of the positive result; the lab does not call positive results to the treatment team. The BPA is displayed in a sidebar on the patient’s EHR home screen and is visible to all members of the care team. The IC precautions implemented at our institution align with the Centers for Disease Control and Prevention’s guideline for isolation precautions and preventing transmission of infectious agents in healthcare settings (Table 1).^10^ Patients with a positive mPCR are placed into private inpatient rooms upon admission. All clinical staff receive education regarding appropriate isolation and personal protective equipment (PPE) requirements during their onboarding process. It is within the scope of RNs to place patients into isolation; however, providers are also entitled to order isolation when they recognize an appropriate indication. The IC team is the final check to ensure that appropriate precautions are placed. The IC team is available on weekdays during daytime hours and provides review of TBPs placed within the previous 24 hours. During the study period, universal masking of healthcare workers during all patient encounters was an expectation due to the COVID-19 pandemic.

**Table 1.** Trinity health grand rapids appropriate infection control precautions

Pathogen	Precautions
Adenovirus	Contact + Droplet
Coronaviruses (not SARS-CoV-2)	Contact + Droplet
Human Metapneumovirus	Contact + Droplet
Influenza A or B	Droplet
Parainfluenza	Contact + Droplet
Rhinovirus/Enterovirus	Droplet
Respiratory Syncytial Virus (RSV)	Contact + Droplet

### Study endpoints

The type of isolation precautions initiated and the time to EHR documenation of precautions after the mPCR resulted were collected. The primary objective was to determine the proportion of patients with appropriate precautions placed within 3 hours of a positive rapid upper respiratory mPCR result for a non-SARS-CoV-2 virus. Three hours was selected as this is the average length of ED stay. Secondary objectives included determining the incidence of over- and under-isolation and evaluating proportion of patients appropriately isolated during their hospital stay if admitted.

### Statistical analysis

A convenience sample of 250 patients was pursued to meet these objectives. Bivariate analyses for categorical data were performed utilizing Chi-square or Fischer’s exact tests, as appropriate. Interval data were compared using the Mann-Whitney U test due to the distribution of the data. All analyses were conducted utilizing SPSS version 22 (Armonk, NY). All statistical tests were 2-tailed and *p* < 0.05 was considered significant.

## Results

In total, 1,185 adult patients were identified as having a mPCR test positive for a viral pathogen within the study period. Patients were screened for inclusion until the desired 250 patient sample size was met. Detection of SARs-CoV-2 was the most common reason for patient exclusion (*n* = 765). Patient and infection characteristics are presented in Table 2; Rhinovirus (41.6%) was the most often identified virus. A total of 120 (48%) patients were treated and discharged from the ED while 130 (52%) were admitted for inpatient care.

**Table 2.** Baseline patient characteristics and clinical presentation

	All patients (*n* = 250)	Appropriate precautions^a^ (*n* = 57)	Inappropriate precautions^b^ (*n* = 193)	*P*-value
**Baseline characteristics**				
Male, *n* (%)	117 (46.8)	25 (43.9)	92 (47.7)	0.613
Age, median [IQR]	61 [43,71]	69 [60,75]	58 [38,69]	**<0.001**
Charlson score, median [IQR]	2 [1,3]	4 [3,7]	3 [1,5]	**<0.001**
Immunosuppressed^c^, *n* (%)	15 (6.0)	8 (14.0)	7 (3.6)	**0.004**
Chronic pulmonary disease, *n* (%)	128 (51.2)	22 (38.6)	106 (54.9)	**0.030**
Smoking history, *n* (%)	159 (63.6)	39 (68.4)	120 (62.2)	0.389
Recent resp tract infection^d^, *n* (%)	21 (8.4)	6 (10.5)	15 (7.8)	0.510
Recent admission^d^, *n* (%)	25 (10.0)	5 (8.8)	20 (10.4)	0.725
**Clinical presentation**				
Febrile, *n* (%)	45 (18.0)	13 (22.8)	32 (16.6)	0.282
Tachypneic, *n* (%)	172 (68.8)	41 (71.9)	131 (67.9)	0.562
Tachycardic, *n* (%)	179 (71.6)	43 (75.4)	136 (70.5)	0.465
Leukocytosis, *n* (%)	41 (16.4)	12 (21.1)	29 (16.4)	0.420
SIRS criteria met, *n* (%)	155 (62.0)	40 (70.2)	115 (59.6)	0.148
Symptoms greater than 7 days, *n* (%)	24 (9.6)	5 (8.8)	19 (9.8)	0.809
Cough, *n* (%)	218 (87.2)	49 (86.0)	169 (87.6)	0.751
Acute shortness of breath, *n* (%)	198 (79.2)	48 (84.2)	150 (77.7)	0.289
Purulent sputum, *n* (%)	52 (20.8)	18 (31.6)	34 (17.6)	**0.022**
Pleuritic chest pain, *n* (%)	66 (26.4)	11 (19.3)	55 (28.5)	0.166
Hemoptysis, *n* (%)	8 (3.2)	2 (3.5)	6 (3.1)	0.880
Multilobar infiltrate on imaging, *n* (%)	23 (9.2)	12 (21.4)	11 (6.2)	**0.001**
Bilateral infiltrate on imaging, *n* (%)	38 (15.2)	13 (23.2)	25 (14.0)	0.105
Unilobar infiltrate on imaging, *n* (%)	31 (12.4)	9 (16.1)	22 (12.4)	0.475
Pleural effusion on imaging, *n* (%)	29 (11.6)	8 (14.3)	21 (11.8)	0.622
**Viral pathogen detected**				
Adenovirus, *n* (%)	6 (2.4)	1 (1.8)	5 (2.6)	–
Coronavirus (no SARS-CoV-2), *n* (%)	32 (12.8)	7 (3.6)	25 (13.0)	–
Human Metapneumovirus, *n* (%)	36 (14.4)	14 (24.6)	22 (11.4)	–
Influenza A, *n* (%)	34 (13.6)	8 (14.0)	26 (13.5)	–
Parainfluenza, *n* (%)	29 (11.6)	8 (14.0)	21 (10.9)	–
Rhinovirus, *n* (%)	104 (41.6)	17 (29.8)	87 (45.1)	–
RSV, *n* (%)	15 (6.0)	2 (3.5)	13 (6.7)	–

a Appropriate precautions (droplet +/− contact) within 3 hours of positive respiratory mPCR test.

b Inappropriate precautions (droplet +/− contact) within 3 hours of positive respiratory mPCR test.

c Receiving chemotherapy, monoclonal antibody, tacrolimus, cyclosporine, mycophenolate, azathioprine, or corticosteroids equivalent to 20 mg of prednisone ≥28 days or 40 mg of prednisone for ≥14 days.

d Within 30 days prior to arrival.

Regarding the primary endpoint, 57 patients (22.8%) had appropriate precautions placed within 3 hours of positive viral mPCR result. Under-isolation was observed in 168 patients (67.2%) due to absence of droplet precautions (34%), absence of contact precautions (4.4%), or absence of both droplet and contact precautions (28.8%). Over-isolation occurred in 25 patients (10%) due to placement of contact precautions when they were not indicated. Patients receiving appropriate precautions were more likely to be older, immunocompromised, and have more comorbidities.

In the subgroup of 130 patients who were admitted for inpatient care, between 3 hours and patient discharge isolation precautions were ordered appropriately for 82 patients (63.1%). Under-isolation was observed in 32 patients (24.7%) due to absence of droplet precautions (7.7%), absence of contact precautions (10.8%), or absence of both droplet and contact precautions (6.2%). Over-isolation occurred in 16 patients (12.3%).

## Discussion

Transmission-based precautions limit the spread of infections to healthcare team members, visitors, and patients.^10^ This study took place when universal masking was required due to the SARS-CoV-2 pandemic, and we found that most patients did not have appropriate isolation precautions ordered within 3 hours of mPCR detection of non-SARS-CoV-2 respiratory viruses. Additionally, of those admitted to the hospital, there were still nearly 40% of patients lacking appropriate precautions during their stay. Infection control efforts at the time were centered around preventing SARS-CoV-2 spread, which shifted resources away from surveillance and education of routine TBPs. Now, post-pandemic with universal masking recommendations removed, these findings demonstrate the need to get back to the basics of IC practices to better protect individuals from viral pathogens. Based on this data, the IC team created a standard work plan to consistently review patient infections and corresponding isolation daily. Further, there will need to be consistent education for all staff members. A key group to target with education is the ED staff as they are often the front-line providers and may be at highest risk for transmission of infection providing evaluation before diagnostic test results are available.

There are some limitations to consider. This was a single-center, retrospective study relying on appropriate documentation in the EHR for accurate data collection. On January 26th, 2020, our institution implemented a new EHR shortly before the emergence of SARS-CoV-2. Limited time to acclimate and train with the EHR could have caused unfamiliarity with EHR features and contributed to low rates of TBP documentation. Healthcare professionals may have been exercising precautions without corresponding documentation once universal masking in healthcare became a requirement. We could not retrospectively identify if appropriate signage and PPE were placed outside of patient rooms when documentation was missing. Additionally, our institution utilizes a passive BPA to identify patients requiring TBPs; however, use of an active BPA is associated with higher compliance to appropriate actions.^11^

## Conclusion

Most patients did not have appropriate TBPs ordered within 3 hours of positive mPCR result for a non-SARS-CoV-2 respiratory virus. With the recent cessation of universal masking, a focus on IC best practices is needed to ensure the safety of healthcare professionals and patients.
